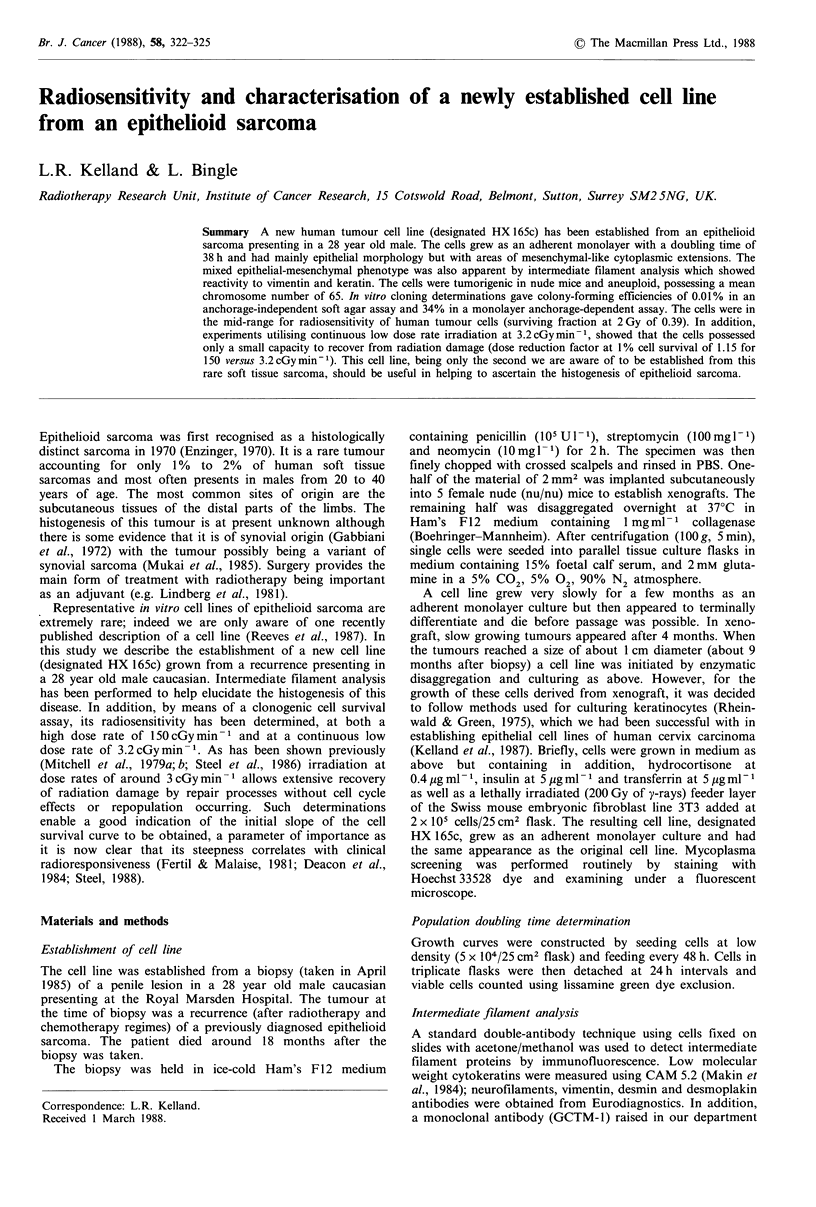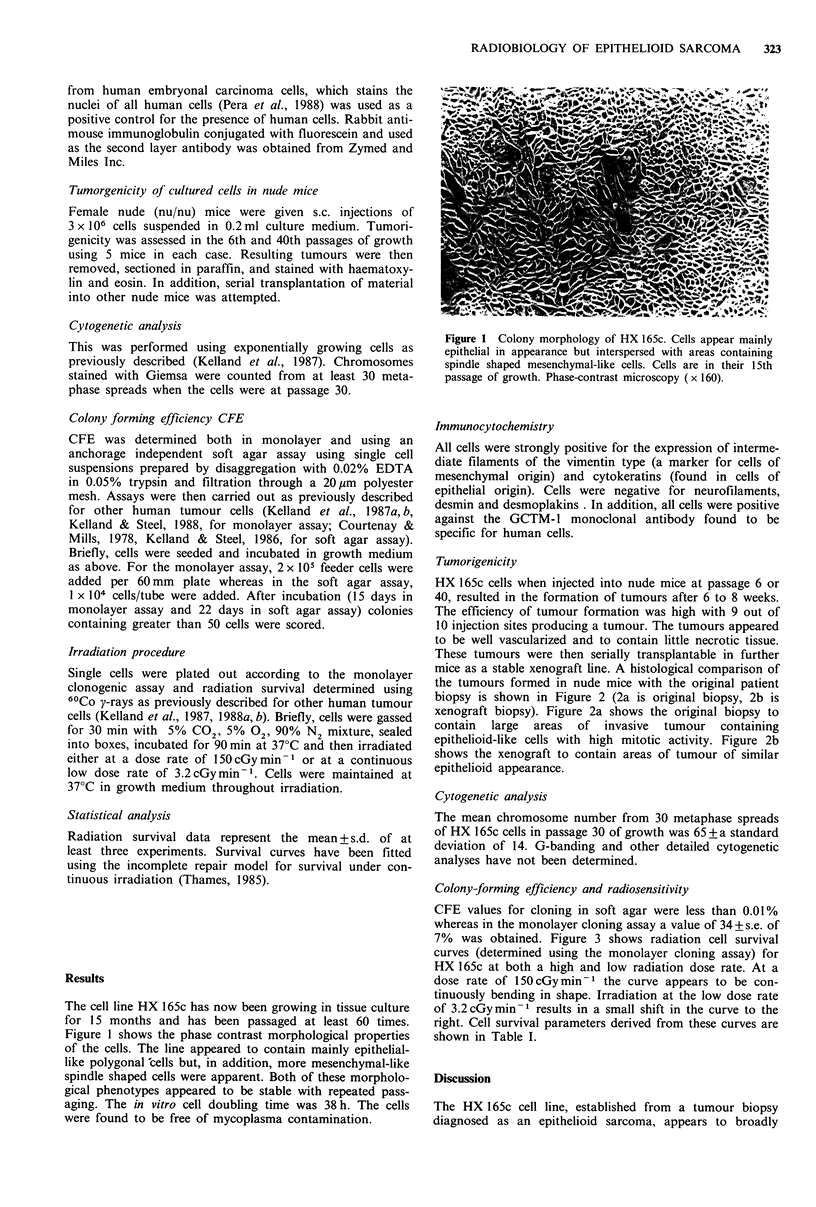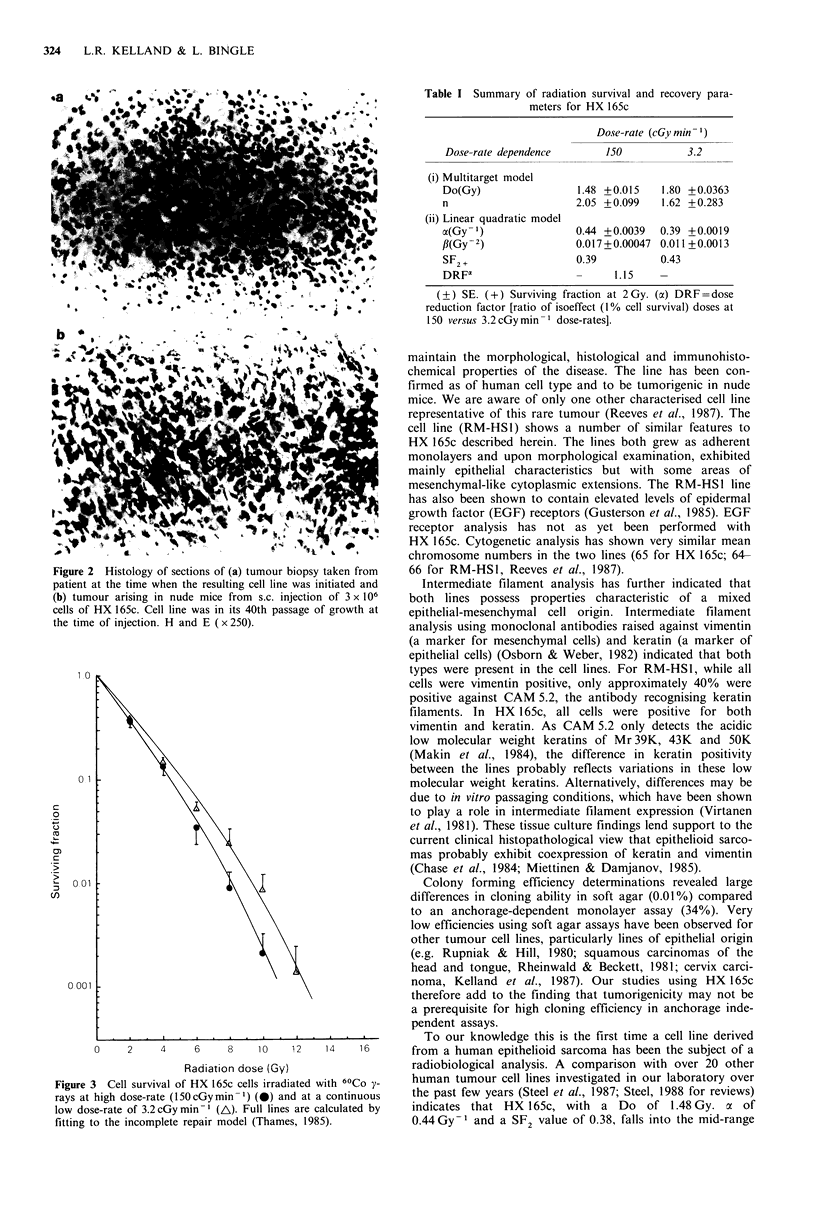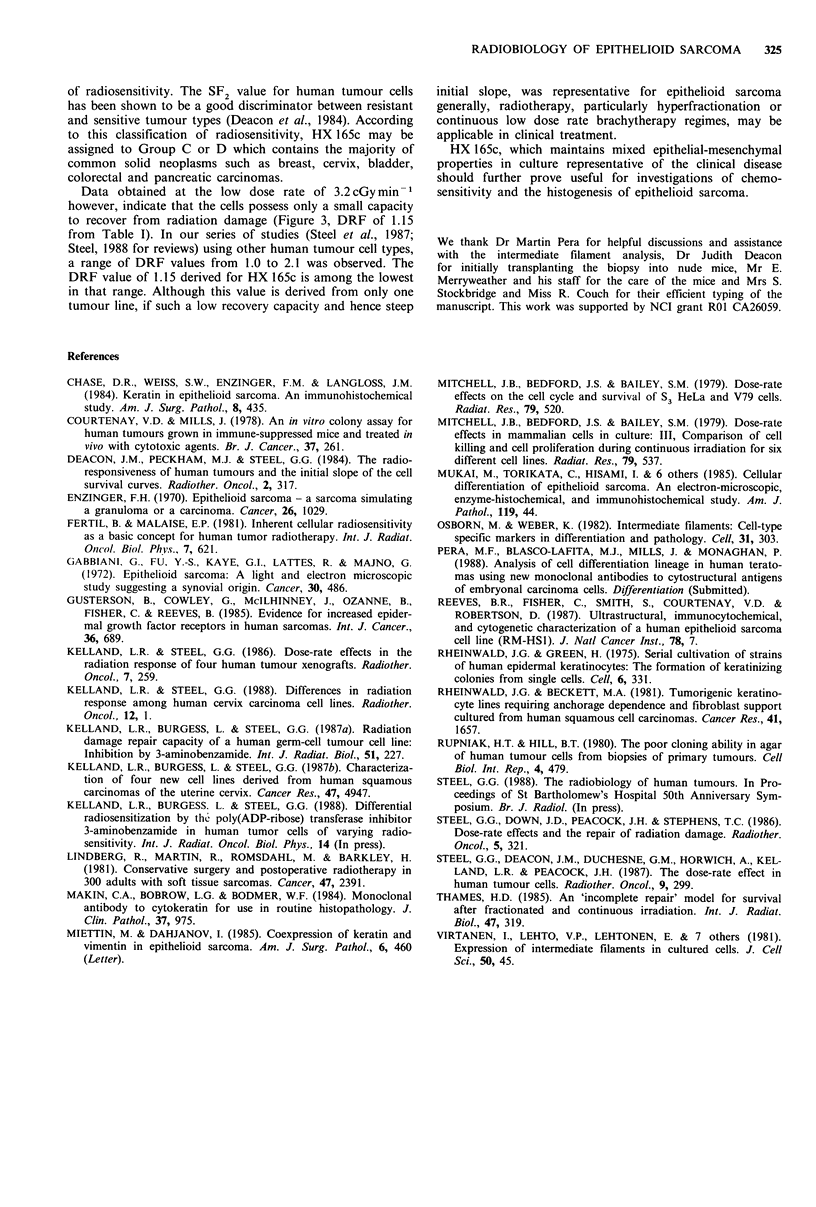# Radiosensitivity and characterisation of a newly established cell line from an epithelioid sarcoma.

**DOI:** 10.1038/bjc.1988.211

**Published:** 1988-09

**Authors:** L. R. Kelland, L. Bingle

**Affiliations:** Radiotherapy Research Unit, Institute of Cancer Research, Sutton, Surrey, UK.

## Abstract

**Images:**


*Br. J. Cancer* (1988), **58**, 322–325 © The Macmillan Press Ltd., 1988

### Radiosensitivity and characterisation of a newly established cell line from an epithelioid sarcoma

L.R. Kelland & L. Bingle

*Radiotherapy Research Unit, Institute of Cancer Research, 15 Cotswold Road, Belmont, Sutton, Surrey SM2 5NG, UK.*

**Summary** A new human tumour cell line (designated HX 165c) has been established from an epithelioid sarcoma presenting in a 28 year old male. The cells grew as an adherent monolayer with a doubling time of 38 h and had mainly epithelial morphology but with areas of mesenchymal-like cytoplasmic extensions. The mixed epithelial-mesenchymal phenotype was also apparent by intermediate filament analysis which showed reactivity to vimentin and keratin. The cells were tumorigenic in nude mice and aneuploid, possessing a mean chromosome number of 65. *In vitro* cloning determinations gave colony-forming efficiencies of 0.01% in an anchorage-independent soft agar assay and 34% in a monolayer anchorage-dependent assay. The cells were in the mid-range for radiosensitivity of human tumour cells (surviving fraction at 2 Gy of 0.39). In addition, experiments utilising continuous low dose rate irradiation at 3.2 cGy $min^{-1}$, showed that the cells possessed only a small capacity to recover from radiation damage (dose reduction factor at 1% cell survival of 1.15 for 150 *versus* 3.2 cGy $min^{-1}$). This cell line, being only the second we are aware of to be established from this rare soft tissue sarcoma, should be useful in helping to ascertain the histogenesis of epithelioid sarcoma.

Epithelioid sarcoma was first recognised as a histologically distinct sarcoma in 1970 (Enzinger, 1970). It is a rare tumour accounting for only 1% to 2% of human soft tissue sarcomas and most often presents in males from 20 to 40 years of age. The most common sites of origin are the subcutaneous tissues of the distal parts of the limbs. The histogenesis of this tumour is at present unknown although there is some evidence that it is of synovial origin (Gabbiani *et al.*, 1972) with the tumour possibly being a variant of synovial sarcoma (Mukai *et al.*, 1985). Surgery provides the main form of treatment with radiotherapy being important as an adjuvant (e.g. Lindberg *et al.*, 1981).

Representative *in vitro* cell lines of epithelioid sarcoma are extremely rare; indeed we are only aware of one recently published description of a cell line (Reeves *et al.*, 1987). In this study we describe the establishment of a new cell line (designated HX 165c) grown from a recurrence presenting in a 28 year old male caucasian. Intermediate filament analysis has been performed to help elucidate the histogenesis of this disease. In addition, by means of a clonogenic cell survival assay, its radiosensitivity has been determined, at both a high dose rate of 150 cGy $min^{-1}$ and at a continuous low dose rate of 3.2 cGy $min^{-1}$. As has been shown previously (Mitchell *et al.*, 1979*a*; *b*; Steel *et al.*, 1986) irradiation at dose rates of around 3 cGy $min^{-1}$ allows extensive recovery of radiation damage by repair processes without cell cycle effects or repopulation occurring. Such determinations enable a good indication of the initial slope of the cell survival curve to be obtained, a parameter of importance as it is now clear that its steepness correlates with clinical radioresponsiveness (Fertil & Malaise, 1981; Deacon *et al.*, 1984; Steel, 1988).

### Materials and methods

### *Establishment of cell line*

The cell line was established from a biopsy (taken in April 1985) of a penile lesion in a 28 year old male caucasian presenting at the Royal Marsden Hospital. The tumour at the time of biopsy was a recurrence (after radiotherapy and chemotherapy regimes) of a previously diagnosed epithelioid sarcoma. The patient died around 18 months after the biopsy was taken.

The biopsy was held in ice-cold Ham's F12 medium containing penicillin ($10^5$ U $l^{-1}$), streptomycin (100 mg $l^{-1}$) and neomycin (10 mg $l^{-1}$) for 2 h. The specimen was then finely chopped with crossed scalpels and rinsed in PBS. One-half of the material of 2 $mm^2$ was implanted subcutaneously into 5 female nude (nu/nu) mice to establish xenografts. The remaining half was disaggregated overnight at 37°C in Ham's F12 medium containing 1 mg $ml^{-1}$ collagenase (Boehringer–Mannheim). After centrifugation (100 *g*, 5 min), single cells were seeded into parallel tissue culture flasks in medium containing 15% foetal calf serum, and 2 mM glutamine in a 5% $CO_2$, 5% $O_2$, 90% $N_2$ atmosphere.

A cell line grew very slowly for a few months as an adherent monolayer culture but then appeared to terminally differentiate and die before passage was possible. In xenograft, slow growing tumours appeared after 4 months. When the tumours reached a size of about 1 cm diameter (about 9 months after biopsy) a cell line was initiated by enzymatic disaggregation and culturing as above. However, for the growth of these cells derived from xenograft, it was decided to follow methods used for culturing keratinocytes (Rheinwald & Green, 1975), which we had been successful with in establishing epithelial cell lines of human cervix carcinoma (Kelland *et al.*, 1987). Briefly, cells were grown in medium as above but containing in addition, hydrocortisone at 0.4 $\mu$g $ml^{-1}$, insulin at 5 $\mu$g $ml^{-1}$ and transferrin at 5 $\mu$g $ml^{-1}$ as well as a lethally irradiated (200 Gy of $\gamma$-rays) feeder layer of the Swiss mouse embryonic fibroblast line 3T3 added at $2 \times 10^5$ cells/25 $cm^2$ flask. The resulting cell line, designated HX 165c, grew as an adherent monolayer culture and had the same appearance as the original cell line. Mycoplasma screening was performed routinely by staining with Hoechst 33528 dye and examining under a fluorescent microscope.

### *Population doubling time determination*

Growth curves were constructed by seeding cells at low density ($5 \times 10^4$/25 $cm^2$ flask) and feeding every 48 h. Cells in triplicate flasks were then detached at 24 h intervals and viable cells counted using lissamine green dye exclusion.

### *Intermediate filament analysis*

A standard double-antibody technique using cells fixed on slides with acetone/methanol was used to detect intermediate filament proteins by immunofluorescence. Low molecular weight cytokeratins were measured using CAM 5.2 (Makin *et al.*, 1984); neurofilaments, vimentin, desmin and desmoplakin antibodies were obtained from Eurodiagnostics. In addition, a monoclonal antibody (GCTM-1) raised in our department

Correspondence: L.R. Kelland.
Received 1 March 1988.

from human embryonal carcinoma cells, which stains the nuclei of all human cells (Pera *et al.*, 1988) was used as a positive control for the presence of human cells. Rabbit anti-mouse immunoglobulin conjugated with fluorescein and used as the second layer antibody was obtained from Zymed and Miles Inc.

*Tumorgenicity of cultured cells in nude mice*

Female nude (nu/nu) mice were given s.c. injections of $3 \times 10^6$ cells suspended in 0.2 ml culture medium. Tumorigenicity was assessed in the 6th and 40th passages of growth using 5 mice in each case. Resulting tumours were then removed, sectioned in paraffin, and stained with haematoxylin and eosin. In addition, serial transplantation of material into other nude mice was attempted.

*Cytogenetic analysis*

This was performed using exponentially growing cells as previously described (Kelland *et al.*, 1987). Chromosomes stained with Giemsa were counted from at least 30 metaphase spreads when the cells were at passage 30.

*Colony forming efficiency CFE*

CFE was determined both in monolayer and using an anchorage independent soft agar assay using single cell suspensions prepared by disaggregation with 0.02% EDTA in 0.05% trypsin and filtration through a 20 $\mu$m polyester mesh. Assays were then carried out as previously described for other human tumour cells (Kelland *et al.*, 1987*a*, *b*, Kelland & Steel, 1988, for monolayer assay; Courtenay & Mills, 1978, Kelland & Steel, 1986, for soft agar assay). Briefly, cells were seeded and incubated in growth medium as above. For the monolayer assay, $2 \times 10^5$ feeder cells were added per 60 mm plate whereas in the soft agar assay, $1 \times 10^4$ cells/tube were added. After incubation (15 days in monolayer assay and 22 days in soft agar assay) colonies containing greater than 50 cells were scored.

*Irradiation procedure*

Single cells were plated out according to the monolayer clonogenic assay and radiation survival determined using $^{60}$Co $\gamma$-rays as previously described for other human tumour cells (Kelland *et al.*, 1987, 1988*a*, *b*). Briefly, cells were gassed for 30 min with 5% $CO_2$, 5% $O_2$, 90% $N_2$ mixture, sealed into boxes, incubated for 90 min at 37°C and then irradiated either at a dose rate of 150 cGy min$^{-1}$ or at a continuous low dose rate of 3.2 cGy min$^{-1}$. Cells were maintained at 37°C in growth medium throughout irradiation.

*Statistical analysis*

Radiation survival data represent the mean $\pm$ s.d. of at least three experiments. Survival curves have been fitted using the incomplete repair model for survival under continuous irradiation (Thames, 1985).

### Results

The cell line HX 165c has now been growing in tissue culture for 15 months and has been passaged at least 60 times. Figure 1 shows the phase contrast morphological properties of the cells. The line appeared to contain mainly epithelial-like polygonal cells but, in addition, more mesenchymal-like spindle shaped cells were apparent. Both of these morphological phenotypes appeared to be stable with repeated passaging. The *in vitro* cell doubling time was 38 h. The cells were found to be free of mycoplasma contamination.

**Figure 1** Colony morphology of HX 165c. Cells appear mainly epithelial in appearance but interspersed with areas containing spindle shaped mesenchymal-like cells. Cells are in their 15th passage of growth. Phase-contrast microscopy ($\times$ 160).

*Immunocytochemistry*

All cells were strongly positive for the expression of intermediate filaments of the vimentin type (a marker for cells of mesenchymal origin) and cytokeratins (found in cells of epithelial origin). Cells were negative for neurofilaments, desmin and desmoplakins . In addition, all cells were positive against the GCTM-1 monoclonal antibody found to be specific for human cells.

*Tumorigenicity*

HX 165c cells when injected into nude mice at passage 6 or 40, resulted in the formation of tumours after 6 to 8 weeks. The efficiency of tumour formation was high with 9 out of 10 injection sites producing a tumour. The tumours appeared to be well vascularized and to contain little necrotic tissue. These tumours were then serially transplantable in further mice as a stable xenograft line. A histological comparison of the tumours formed in nude mice with the original patient biopsy is shown in Figure 2 (2a is original biopsy, 2b is xenograft biopsy). Figure 2a shows the original biopsy to contain large areas of invasive tumour containing epithelioid-like cells with high mitotic activity. Figure 2b shows the xenograft to contain areas of tumour of similar epithelioid appearance.

*Cytogenetic analysis*

The mean chromosome number from 30 metaphase spreads of HX 165c cells in passage 30 of growth was 65 $\pm$ a standard deviation of 14. G-banding and other detailed cytogenetic analyses have not been determined.

*Colony-forming efficiency and radiosensitivity*

CFE values for cloning in soft agar were less than 0.01% whereas in the monolayer cloning assay a value of 34 $\pm$ s.e. of 7% was obtained. Figure 3 shows radiation cell survival curves (determined using the monolayer cloning assay) for HX 165c at both a high and low radiation dose rate. At a dose rate of 150 cGy min$^{-1}$ the curve appears to be continuously bending in shape. Irradiation at the low dose rate of 3.2 cGy min$^{-1}$ results in a small shift in the curve to the right. Cell survival parameters derived from these curves are shown in Table I.

### Discussion

The HX 165c cell line, established from a tumour biopsy diagnosed as an epithelioid sarcoma, appears to broadly

**Figure 2** Histology of sections of (**a**) tumour biopsy taken from patient at the time when the resulting cell line was initiated and (**b**) tumour arising in nude mice from s.c. injection of $3 \times 10^6$ cells of HX 165c. Cell line was in its 40th passage of growth at the time of injection. H and E ($\times 250$).

**Figure 3** Cell survival of HX 165c cells irradiated with $^{60}Co$ $\gamma$-rays at high dose-rate (150 cGy min$^{-1}$) (●) and at a continuous low dose-rate of 3.2 cGy min$^{-1}$ (△). Full lines are calculated by fitting to the incomplete repair model (Thames, 1985).

**Table I** Summary of radiation survival and recovery parameters for HX 165c

| | *Dose-rate* (*cGy min*$^{-1}$) | |
|---|---|---|
| *Dose-rate dependence* | *150* | *3.2* |
| (i) Multitarget model | | |
| Do(Gy) | 1.48 ±0.015 | 1.80 ±0.0363 |
| n | 2.05 ±0.099 | 1.62 ±0.283 |
| (ii) Linear quadratic model | | |
| $\alpha$(Gy$^{-1}$) | 0.44 ±0.0039 | 0.39 ±0.0019 |
| $\beta$(Gy$^{-2}$) | 0.017±0.00047 | 0.011±0.0013 |
| $SF_{2+}$ | 0.39 | 0.43 |
| DRF$^{\alpha}$ | – 1.15 | – |

(±) SE. (+) Surviving fraction at 2 Gy. ($\alpha$) DRF = dose reduction factor [ratio of isoeffect (1% cell survival) doses at 150 *versus* 3.2 cGy min$^{-1}$ dose-rates].

maintain the morphological, histological and immunohistochemical properties of the disease. The line has been confirmed as of human cell type and to be tumorigenic in nude mice. We are aware of only one other characterised cell line representative of this rare tumour (Reeves *et al.*, 1987). The cell line (RM-HS1) shows a number of similar features to HX 165c described herein. The lines both grew as adherent monolayers and upon morphological examination, exhibited mainly epithelial characteristics but with some areas of mesenchymal-like cytoplasmic extensions. The RM-HS1 line has also been shown to contain elevated levels of epidermal growth factor (EGF) receptors (Gusterson *et al.*, 1985). EGF receptor analysis has not as yet been performed with HX 165c. Cytogenetic analysis has shown very similar mean chromosome numbers in the two lines (65 for HX 165c; 64–66 for RM-HS1, Reeves *et al.*, 1987).

Intermediate filament analysis has further indicated that both lines possess properties characteristic of a mixed epithelial-mesenchymal cell origin. Intermediate filament analysis using monoclonal antibodies raised against vimentin (a marker for mesenchymal cells) and keratin (a marker of epithelial cells) (Osborn & Weber, 1982) indicated that both types were present in the cell lines. For RM-HS1, while all cells were vimentin positive, only approximately 40% were positive against CAM 5.2, the antibody recognising keratin filaments. In HX 165c, all cells were positive for both vimentin and keratin. As CAM 5.2 only detects the acidic low molecular weight keratins of Mr 39K, 43K and 50K (Makin *et al.*, 1984), the difference in keratin positivity between the lines probably reflects variations in these low molecular weight keratins. Alternatively, differences may be due to *in vitro* passaging conditions, which have been shown to play a role in intermediate filament expression (Virtanen *et al.*, 1981). These tissue culture findings lend support to the current clinical histopathological view that epithelioid sarcomas probably exhibit coexpression of keratin and vimentin (Chase *et al.*, 1984; Miettinen & Damjanov, 1985).

Colony forming efficiency determinations revealed large differences in cloning ability in soft agar (0.01%) compared to an anchorage-dependent monolayer assay (34%). Very low efficiencies using soft agar assays have been observed for other tumour cell lines, particularly lines of epithelial origin (e.g. Rupniak & Hill, 1980; squamous carcinomas of the head and tongue, Rheinwald & Beckett, 1981; cervix carcinoma, Kelland *et al.*, 1987). Our studies using HX 165c therefore add to the finding that tumorigenicity may not be a prerequisite for high cloning efficiency in anchorage independent assays.

To our knowledge this is the first time a cell line derived from a human epithelioid sarcoma has been the subject of a radiobiological analysis. A comparison with over 20 other human tumour cell lines investigated in our laboratory over the past few years (Steel *et al.*, 1987; Steel, 1988 for reviews) indicates that HX 165c, with a Do of 1.48 Gy. $\alpha$ of 0.44 Gy$^{-1}$ and a $SF_2$ value of 0.38, falls into the mid-range of radiosensitivity. The $SF_2$ value for human tumour cells has been shown to be a good discriminator between resistant and sensitive tumour types (Deacon *et al.*, 1984). According to this classification of radiosensitivity, HX 165c may be assigned to Group C or D which contains the majority of common solid neoplasms such as breast, cervix, bladder, colorectal and pancreatic carcinomas.

Data obtained at the low dose rate of $3.2\,cGy\,min^{-1}$ however, indicate that the cells possess only a small capacity to recover from radiation damage (Figure 3, DRF of 1.15 from Table I). In our series of studies (Steel *et al.*, 1987; Steel, 1988 for reviews) using other human tumour cell types, a range of DRF values from 1.0 to 2.1 was observed. The DRF value of 1.15 derived for HX 165c is among the lowest in that range. Although this value is derived from only one tumour line, if such a low recovery capacity and hence steep initial slope, was representative for epithelioid sarcoma generally, radiotherapy, particularly hyperfractionation or continuous low dose rate brachytherapy regimes, may be applicable in clinical treatment.

HX 165c, which maintains mixed epithelial-mesenchymal properties in culture representative of the clinical disease should further prove useful for investigations of chemosensitivity and the histogenesis of epithelioid sarcoma.

We thank Dr Martin Pera for helpful discussions and assistance with the intermediate filament analysis, Dr Judith Deacon for initially transplanting the biopsy into nude mice, Mr E. Merryweather and his staff for the care of the mice and Mrs S. Stockbridge and Miss R. Couch for their efficient typing of the manuscript. This work was supported by NCI grant R01 CA26059.

### References

CHASE, D.R., WEISS, S.W., ENZINGER, F.M. & LANGLOSS, J.M. (1984). Keratin in epithelioid sarcoma. An immunohistochemical study. *Am. J. Surg. Pathol.*, **8**, 435.

COURTENAY, V.D. & MILLS, J. (1978). An *in vitro* colony assay for human tumours grown in immune-suppressed mice and treated *in vivo* with cytotoxic agents. *Br. J. Cancer.*, **37**, 261.

DEACON, J.M., PECKHAM, M.J. & STEEL, G.G. (1984). The radioresponsiveness of human tumours and the initial slope of the cell survival curves. *Radiother. Oncol.*, **2**, 317.

ENZINGER, F.H. (1970). Epithelioid sarcoma – a sarcoma simulating a granuloma or a carcinoma. *Cancer*, **26**, 1029.

FERTIL, B. & MALAISE, E.P. (1981). Inherent cellular radiosensitivity as a basic concept for human tumor radiotherapy. *Int. J. Radiat. Oncol. Biol. Phys.*, **7**, 621.

GABBIANI, G., FU, Y.-S., KAYE, G.I., LATTES, R. & MAJNO, G. (1972). Epithelioid sarcoma: A light and electron microscopic study suggesting a synovial origin. *Cancer*, **30**, 486.

GUSTERSON, B., COWLEY, G., McILHINNEY, J., OZANNE, B., FISHER, C. & REEVES, B. (1985). Evidence for increased epidermal growth factor receptors in human sarcomas. *Int. J. Cancer.*, **36**, 689.

KELLAND, L.R. & STEEL, G.G. (1986). Dose-rate effects in the radiation response of four human tumour xenografts. *Radiother. Oncol.*, **7**, 259.

KELLAND, L.R. & STEEL, G.G. (1988). Differences in radiation response among human cervix carcinoma cell lines. *Radiother. Oncol.*, **12**, 1.

KELLAND, L.R., BURGESS, L. & STEEL, G.G. (1987*a*). Radiation damage repair capacity of a human germ-cell tumour cell line: Inhibition by 3-aminobenzamide. *Int. J. Radiat. Biol.*, **51**, 227.

KELLAND, L.R., BURGESS, L. & STEEL, G.G. (1987*b*). Characterization of four new cell lines derived from human squamous carcinomas of the uterine cervix. *Cancer Res.*, **47**, 4947.

KELLAND, L.R., BURGESS, L. & STEEL, G.G. (1988). Differential radiosensitization by the poly(ADP-ribose) transferase inhibitor 3-aminobenzamide in human tumor cells of varying radiosensitivity. *Int. J. Radiat. Oncol. Biol. Phys.*, **14** (In press).

LINDBERG, R., MARTIN, R., ROMSDAHL, M. & BARKLEY, H. (1981). Conservative surgery and postoperative radiotherapy in 300 adults with soft tissue sarcomas. *Cancer*, **47**, 2391.

MAKIN, C.A., BOBROW, L.G. & BODMER, W.F. (1984). Monoclonal antibody to cytokeratin for use in routine histopathology. *J. Clin. Pathol.*, **37**, 975.

MIETTIN, M. & DAHJANOV, I. (1985). Coexpression of keratin and vimentin in epithelioid sarcoma. *Am. J. Surg. Pathol.*, **6**, 460 (*Letter*).

MITCHELL, J.B., BEDFORD, J.S. & BAILEY, S.M. (1979). Dose-rate effects on the cell cycle and survival of $S_3$ HeLa and V79 cells. *Radiat. Res.*, **79**, 520.

MITCHELL, J.B., BEDFORD, J.S. & BAILEY, S.M. (1979). Dose-rate effects in mammalian cells in culture: III, Comparison of cell killing and cell proliferation during continuous irradiation for six different cell lines. *Radiat. Res.*, **79**, 537.

MUKAI, M., TORIKATA, C., HISAMI, I. & 6 others (1985). Cellular differentiation of epithelioid sarcoma. An electron-microscopic, enzyme-histochemical, and immunohistochemical study. *Am. J. Pathol.*, **119**, 44.

OSBORN, M. & WEBER, K. (1982). Intermediate filaments: Cell-type specific markers in differentiation and pathology. *Cell*, **31**, 303.

PERA, M.F., BLASCO-LAFITA, M.J., MILLS, J. & MONAGHAN, P. (1988). Analysis of cell differentiation lineage in human teratomas using new monoclonal antibodies to cytostructural antigens of embryonal carcinoma cells. *Differentiation* (Submitted).

REEVES, B.R., FISHER, C., SMITH, S., COURTENAY, V.D. & ROBERTSON, D. (1987). Ultrastructural, immunocytochemical, and cytogenetic characterization of a human epithelioid sarcoma cell line (RM-HS1). *J. Natl Cancer Inst.*, **78**, 7.

RHEINWALD, J.G. & GREEN, H. (1975). Serial cultivation of strains of human epidermal keratinocytes: The formation of keratinizing colonies from single cells. *Cell*, **6**, 331.

RHEINWALD, J.G. & BECKETT, M.A. (1981). Tumorigenic keratinocyte lines requiring anchorage dependence and fibroblast support cultured from human squamous cell carcinomas. *Cancer Res.*, **41**, 1657.

RUPNIAK, H.T. & HILL, B.T. (1980). The poor cloning ability in agar of human tumour cells from biopsies of primary tumours. *Cell Biol. Int. Rep.*, **4**, 479.

STEEL, G.G. (1988). The radiobiology of human tumours. In Proceedings of St Bartholomew's Hospital 50th Anniversary Symposium. *Br. J. Radiol.* (In press).

STEEL, G.G., DOWN, J.D., PEACOCK, J.H. & STEPHENS, T.C. (1986). Dose-rate effects and the repair of radiation damage. *Radiother. Oncol.*, **5**, 321.

STEEL, G.G., DEACON, J.M., DUCHESNE, G.M., HORWICH, A., KELLAND, L.R. & PEACOCK, J.H. (1987). The dose-rate effect in human tumour cells. *Radiother. Oncol.*, **9**, 299.

THAMES, H.D. (1985). An 'incomplete repair' model for survival after fractionated and continuous irradiation. *Int. J. Radiat. Biol.*, **47**, 319.

VIRTANEN, I., LEHTO, V.P., LEHTONEN, E. & 7 others (1981). Expression of intermediate filaments in cultured cells. *J. Cell Sci.*, **50**, 45.